# Genome-Wide Association Study Reveals Novel Genes Associated with Culm Cellulose Content in Bread Wheat (*Triticum aestivum*, L.)

**DOI:** 10.3389/fpls.2017.01913

**Published:** 2017-11-06

**Authors:** Simerjeet Kaur, Xu Zhang, Amita Mohan, Haixiao Dong, Prashant Vikram, Sukhwinder Singh, Zhiwu Zhang, Kulvinder S. Gill, Kanwarpal S. Dhugga, Jaswinder Singh

**Affiliations:** ^1^Department of Plant Science, McGill University, Sainte Anne de Bellevue, QC, Canada; ^2^Department of Crop and Soil Science, Washington State University, Pullman, WA, United States; ^3^International Maize and Wheat Improvement Center (CIMMYT), El Batán, Texcoco, Mexico

**Keywords:** GWAS, cellulose, wheat, cereals, grasses, SNPs, bioenergy, cell wall

## Abstract

Plant cell wall formation is a complex, coordinated and developmentally regulated process. Cellulose is the most dominant constituent of plant cell walls. Because of its paracrystalline structure, cellulose is the main determinant of mechanical strength of plant tissues. As the most abundant polysaccharide on earth, it is also the focus of cellulosic biofuel industry. To reduce culm lodging in wheat and for improved ethanol production, delineation of the variation for stem cellulose content could prove useful. We present results on the analysis of the stem cellulose content of 288 diverse wheat accessions and its genome-wide association study (GWAS). Cellulose concentration ranged from 35 to 52% (w/w). Cellulose content was normally distributed in the accessions around a mean and median of 45% (w/w). Genome-wide marker-trait association study using 21,073 SNPs helped identify nine SNPs that were associated (*p* < 1E-05) with cellulose content. Four strongly associated (*p* < 8.17E-05) SNP markers were linked to wheat unigenes, which included β*-tubulin, Auxin-induced protein 5NG4*, and a putative transmembrane protein of unknown function. These genes may be directly or indirectly involved in the formation of cellulose in wheat culms. GWAS results from this study have the potential for genetic manipulation of cellulose content in bread wheat and other small grain cereals to enhance culm strength and improve biofuel production.

## Introduction

Increasing world population demands a sustainable increase in the production of food, feed and fuel crops (Scholey et al., 2016). Bread wheat (*Triticum aestivum*) occupies more agricultural area than any other food crop worldwide (http://www.wheatinitiative.org/). In addition to grain production, the annual worldwide production of wheat straw is around 3.5 × 10^8^ tons, which is used as cattle fodder in developing countries and is a potential feedstock for cellulosic ethanol production (Singhania et al., 2014). Wheat straw, which is comprised of cellulose (~40%), hemicelluloses (~35%), and lignin (~25%), is one of the most abundant lignocellulosic raw materials in the world (Ruiz et al., 2013). Cellulose, a paracrystalline polysaccharide, is the main determinant of mechanical strength, which has implications in crop lodging, biotic and abiotic stresses. Cellulose amount in a unit length of the stem explains most of the variation in mechanical strength (Appenzeller et al., 2004; Dhugga, 2007). The proportion of cellulose in the cell wall also affects the total sugar release during the process of enzymatic hydrolysis (Fan et al., 2012; Lindedam et al., 2012). An understanding of the natural variability of cellulose in plants and its association with chromosomal regions could provide markers for enhancing grain and biomass yield (Ciesielski et al., 2014).

Cellulose consists of linear chains of β (1→4) linked glucan (polyglucose) known to be synthesized by the members of superfamily *Glycosyltransferase 2* (*GT2*) called *Cellulose synthase A* (*CesA*; Fujii et al., 2010; Kumar et al., 2016). Twenty-two *CesA* genes have been reported in hexaploid wheat (Kaur et al., 2016). In addition to the *CesA* genes, the *Glycosylhydrolase 9* (*GH9*) family genes are known to have an impact on the synthesis of cellulose in plants (Kotake et al., 2011). Based on the mutant analysis in Arabidopsis, a member of *GH9* family called *KORRIGAN1* (*KOR1*) has been reported to be involved in cellulose synthesis, cell expansion and intracellular trafficking of cellulose synthase complex (CSC; Szyjanowicz et al., 2004; Lei et al., 2014; Vain et al., 2014). Investigation of *brittle culm 1* mutants in rice and *brittle stalk 2* mutant in maize revealed the involvement of COBRA-like proteins in cellulose formation in secondary walls (Ching et al., 2006). Involvement of *Sucrose synthase* (*SuSy*) in channeling substrate to cellulose synthase has also been reported (Fujii et al., 2010). Similarly, several other proteins affect cellulose synthesis, including *chitinase-like 1* (*CSI1*; Sánchez-Rodríguez et al., 2012), *companion of cellulose synthase* (*CC*; Endler et al., 2015), and *tracheary element differentiation-related* (*TED*) 6 and 7 (Rejab et al., 2015).

Variation for the proportion of cellulose in cell wall among wheat varieties is not yet known. This study was planned to identify the genomic regions affecting the variability of cellulose content among diverse spring wheat genotypes through GWAS.

Genes associated with cell wall have been previously explored through GWAS in miscanthus (Slavov et al., 2014), poplar (Porth et al., 2013), maize (Li et al., 2016), and barley (Houston et al., 2015). In barley, genes of *Glycosyltransferase 2* and *Glycosylhydrolase* families were associated with culm cellulose variation. However, none of the genes found in maize through GWAS of stalk cellulose content was specifically involved in the cellulose biosynthetic pathway. In the present study, the stem internodes of 288 spring wheat varieties were analyzed for variation in cellulose content. Utilizing the 21,073 SNPs generated by DArT-seq GBS and cellulosic content, GWAS was performed using fixed and random model circulating probability unification (FarmCPU) method (Liu et al., 2016). Genes, which were not reported previously for their role in cellulose formation, were identified as associated with the culm cellulose content. Gene-trait associations identified in this study might be useful in altering the lignocellulose composition of wheat and other grasses.

## Materials and methods

### Plant material

A worldwide collection of 288 diverse spring wheat germplasm was used for the phenotypic and genotypic analysis. The collection included cultivars from different regions of United States, the International Maize and Wheat Improvement Centre (CIMMYT), Mexico, and historical lines dating back to 1871 (Mohan et al., 2013). The wide span of our collection was intended to capture the maximum variation possible while maintaining a manageable population size. This worldwide collection also represents the various market classes of wheat based on the kernel color, hardiness, and shape. The following types of genotypes were represented based on kernel type: soft white spring (SWS), soft red spring (SRS), hard red spring (HRS), hard white spring (HWS), and club wheat (Mohan et al., 2013). The plants were grown in the greenhouse of the Plant Growth Facilities, Washington State University, Pullman at 22°C/18°C temperature and 16/8 h day/night in 2014-15. Seeds were planted in a randomized design to accommodate the effect of light.

### Phenotypic analysis

The analysis on percent cellulose was performed for 288 diverse spring wheat genotypes, with three replicates per genotype. The first internode (from the base) of the main tiller of each of three mature plants was dried at 80°C. Measured amount of dried sample (45–55 mg) was placed into a pre-weighed 2 ml Eppendorf tubes with a screw cap. A mixture of acetic acid: water: nitric acid (8:2:1) was added to each tube (1.5 ml) and vortexed (Updegraff, 1969). All the tubes were transferred to a steel rack and placed in a boiling water bath for 4 h. The tubes were allowed to cool at room temperature and centrifuged in a swing-out rotor at 10,000 rpm for 10 min. The supernatant was aspirated off, the pellet washed with distilled water four times and finally washed with 90% ethanol. After each wash, the tubes were vortexed and centrifuged at 10,000 rpm for 10 min. The tubes were dried at 80°C followed by determination of percent cellulose on dry matter basis.

### Population structure and GWAS analysis

Principal component analysis (PCA) was used to infer population structure through Genomic Association and Prediction Integrated Tool (GAPIT; Lipka et al., 2012; Ahmad et al., 2015; Tang et al., 2016). Twenty-one thousand and seventy-three SNP markers were obtained by analyzing the genomic DNA with a Genotyping-by-Sequencing (GBS) approach (Mohan et al. unpublished). In brief, genotyping was carried out at DArT Pyt Ltd in Canberra-Australia, using a combination of HiSeq 2000 (Illumina) next-generation sequencing with DArT-seq GBS technology (called DArTseq™). This method follows two-step complexity reductions by using two enzymes, PstI/HpaII and PstI/HhaI, along-with TaqI restriction enzyme to eliminate subsets of PstI -HpaII and PstI-HhaI fragments, respectively. The pooled barcoaded samples were run in a single lane on an Illumina Hiseq 2000 instrument for sequencing. A proprietary analytical pipeline developed by DArT Pyt Ltd was used to obtain the DArT score and SNP tables (http://www.diversityarrays.com/). Fixed and Random Model Circulating Probability Unification (FarmCPU; Liu et al., 2016) in R version 2.15.3 was used to calculate *P*-values for Manhattan Plot and Q-Q plots. A Manhattan plot was generated using the −log10(*p*) values for each SNP with 1% Bonferroni test threshold (Team, 2014). The significance of the genome-wide association between SNP marker and cellulose content was tested at FDR *p* < 0.001.

### Gene annotation

The sequences containing the SNPs were mapped against wheat unigenes downloaded from the NCBI database. Significant SNPs with associated unigenes were annotated using BLASTN with the International Wheat Genome Sequencing Consortium (IWGSC; Mayer et al., 2014) reference Sequence v1.0 (https://www.wheatgenome.org) posted on May 30, 2017. The functions of associated unigenes were also searched with BLASTN through identification of orthologs from other plant species.

## Results

### Cellulose content

**Figure 1:**
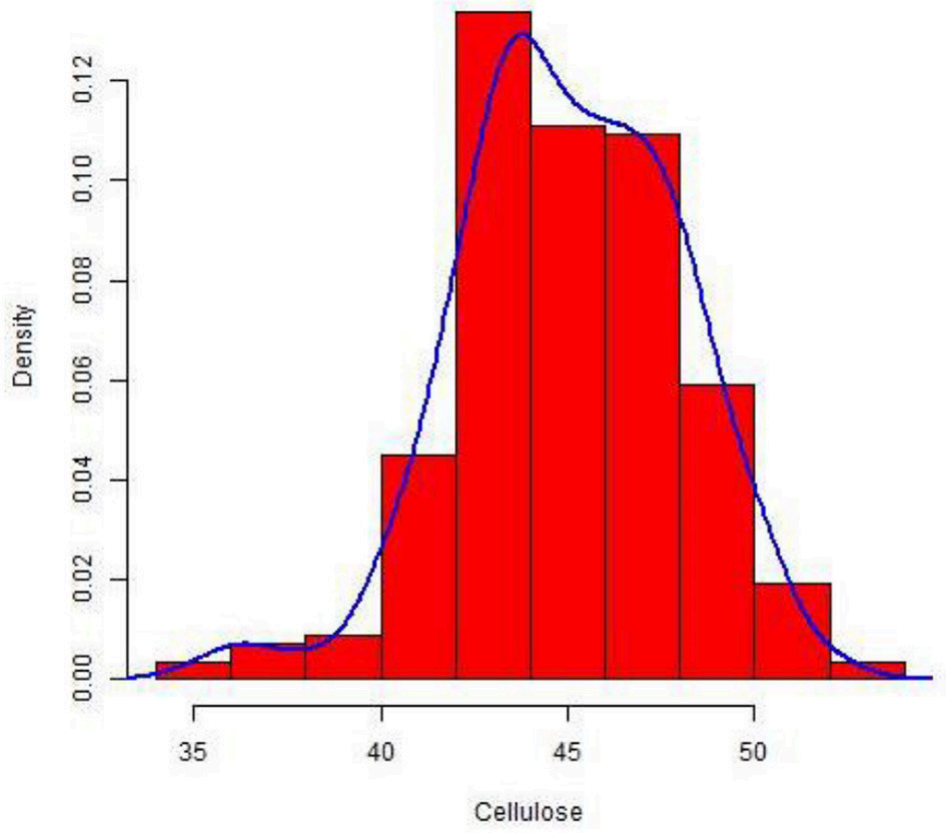
Density plot of percent cellulose among 288 diverse spring wheat accessions.

The culm cellulose content differed significantly in a set of 228 wheat lines with a range of 0.32–0.52 mg and an average of 0.45 mg cellulose/mg of dry weight (Table S2). The cellulose concentration was normally distributed around the mean in the set as depicted in the density plot (Figure 1). As expected from this plot, the calculated median was also similar to the overall mean across the population.

### Principal component analysis and marker-trait associations

**Figure 2:**
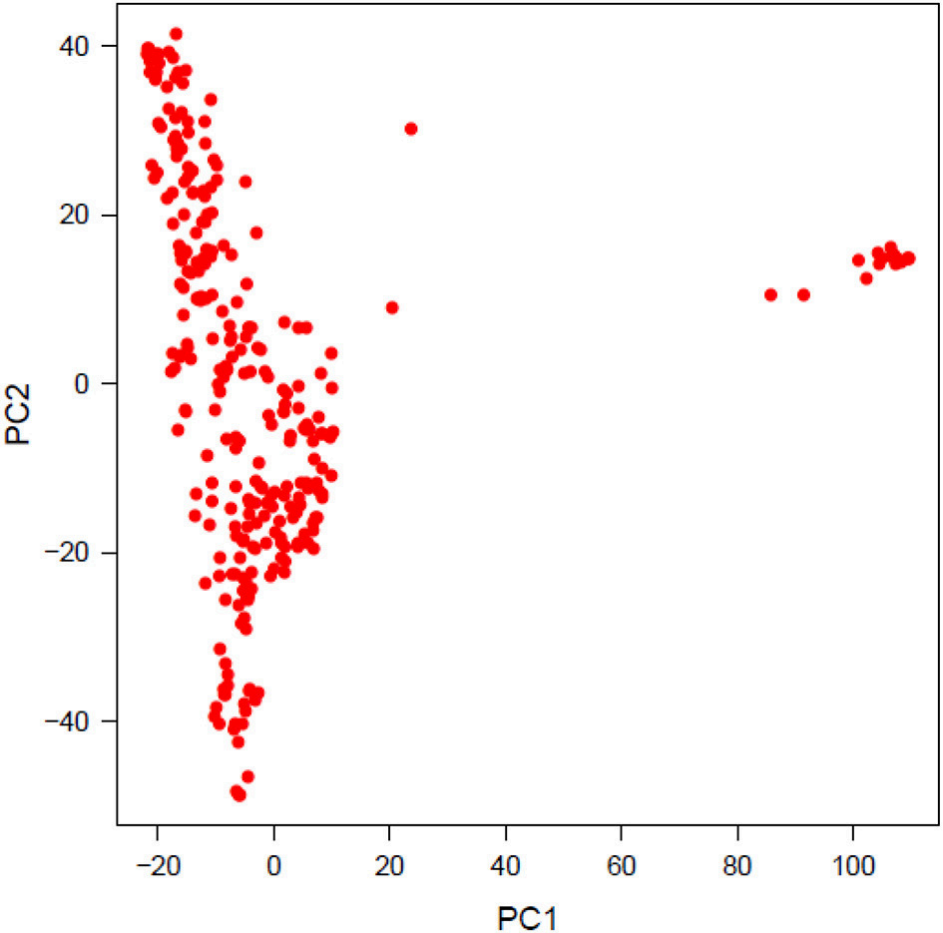
Principal component analysis of 288 diverse genotypes used for GWAS.

PCA was performed to investigate population structure. The first two PCs explained 8.13 and 4.90% variation in the population of lines. Plotting PC2 against PC1 revealed two distinct, a major and a minor, clusters. The minor cluster containing 20 genotypes was removed from the final analysis to account for population structure and the first PC was used as a covariate for GWAS analyses (Figure 2).

**Figure 3:**
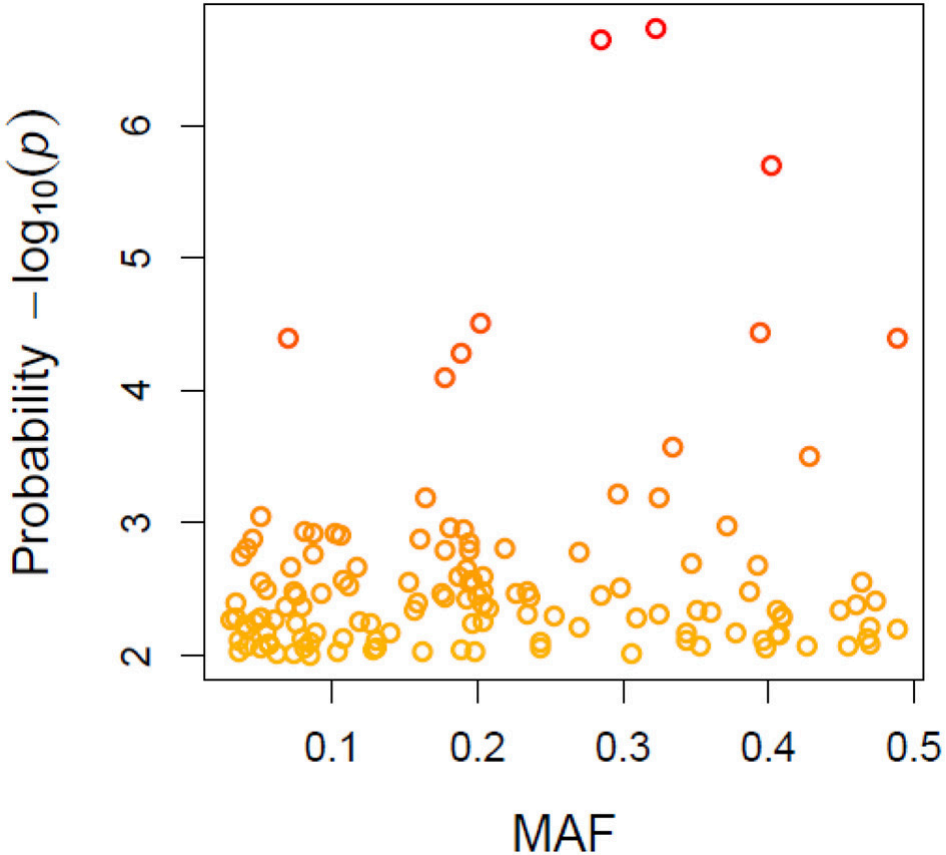
Minor allele frequency (MAF) patterns relative to allele calls for wheat genotypes based on 21073 SNPs.

**Figure 4:**
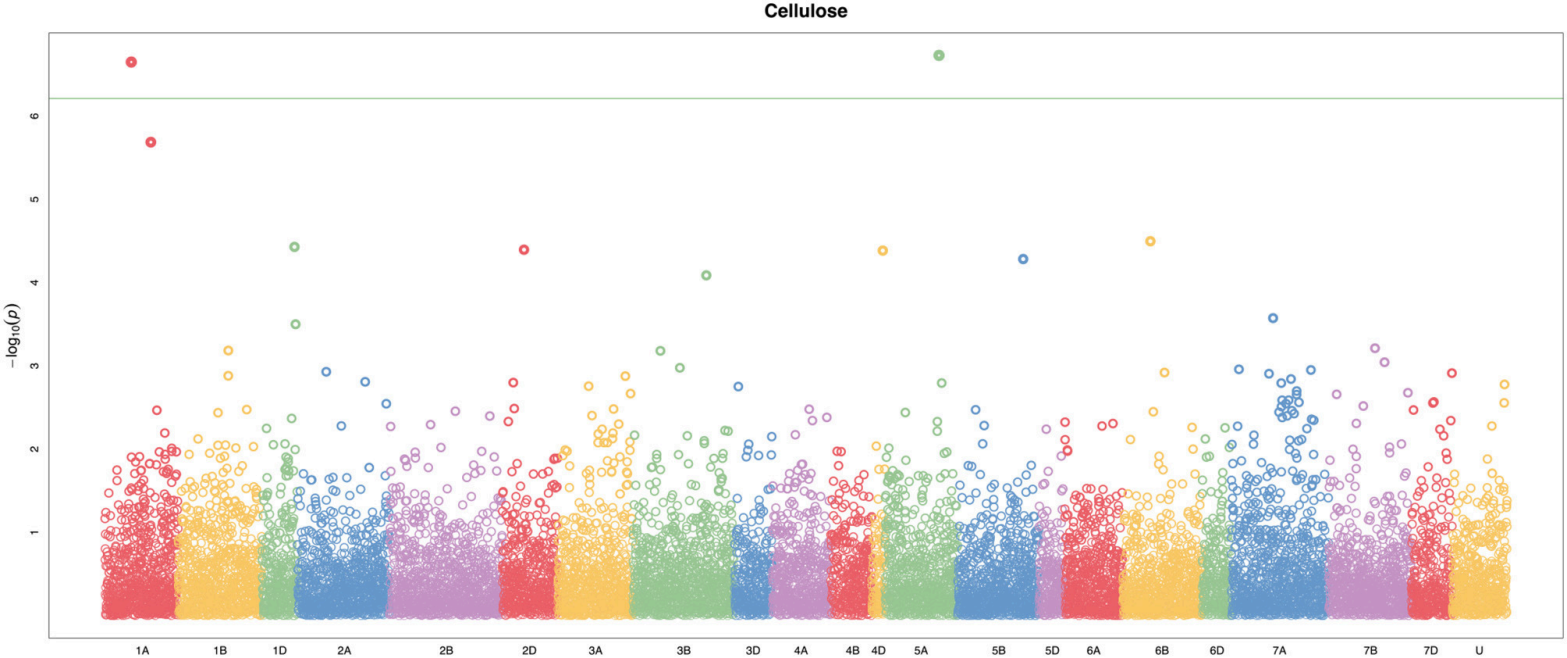
Manhattan plot of genome-wide association study (GWAS) on stem cellulose content (mg cellulose/mg dry weight) by using the FarmCPU. The −log10(*p*–values) from GWAS are plotted against the position on each of the 21 bread wheat chromosomes. U represents unassigned chromosome scaffolds. Two loci on chromosomes 1A and 5A were identified above the Bonferroni threshold correcting for genome-wide multiple tests at type I error of 0.001 (green line).

**Figure 5:**
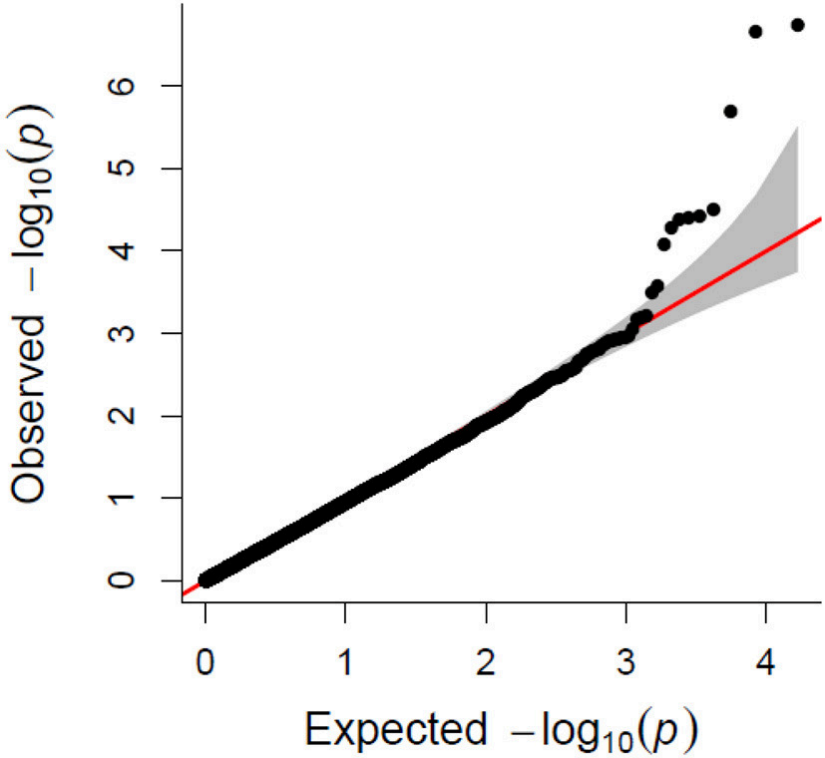
Quantile-quantile (QQ) plot showing the deviation from null hypothesis for associated SNP makers.

A total of 21,073 single nucleotide polymorphic (SNP) markers with a minor allele frequency (MAF) above 5% (Figure 3) and the cellulose content data from 268 lines were subjected to GWAS analysis, which revealed nine significant marker-trait associations with *p*-values of less than 1E-05 (Figure 4). The most significant SNP marker in our analysis corresponded to wheat chromosome 5AL with a *p*-value of 1.86E-07. The second most significant SNP was located on chromosome 1AL with a *p*-value of 2.24E-07. In addition, we found significant SNPs corresponding to chromosome 1AL, 6BS, 1DL, 2DS, 4DL, 5BL, and 3B with *p*-values of less than 1E-05 (Table 1). The quantile-quantile (QQ) plot drawn for calculated *p*-values was used to check spurious associations. The deviation of relatively a few markers from null expectations in the QQ plot supports the significant associations we have identified (Figure 5).

**Table 1:** Regions of wheat genome showing significant associations with stem cellulose content variation based on GWAS.

**SNP ID**	**Allele**	**CHR**	**Scaffold:Position**	***P-*value**	**MAF**	**Unigene**	**Candidate annotation**	**Gene ID (Ensembl)**
1096787|F|040	C>T	5AL	376159:25309	1.86E-07	0.323	gnl|UG|Ta#S13258805	Uncharacterized gene	TRIAE_CS42_5AL_TGACv1_376159_AA1232950S
1018641|F|062	T>C	1AL	138:45403	2.24E-07	0.285	N/A		
100315676|F|050	T>C	1AL	1074:43532	2.05E-06	0.402	gnl|UG|Ta#S52545076	*Tubulin* β*-1* chain	TRIUR3_05395
1080815|F|044	T>C	6BS	514572:36113	3.18E-05	0.202	N/A		
3026141|F|05	A>C	1DL	63549:20036	3.72E-05	0.394	N/A		
1018617|F|035	C>T	2DS	179544:14866	4.02E-05	0.489	gnl|UG|Ta#S65598833	Auxin-induced protein *5NG4*	TRIAE_CS42_2DS_TGACv1_179544_AA0607850
1245047|F|039	C>T	4DL	344580:40916	4.12E-05	0.070	N/A		
1069330|F|06	T>A	5BL	406565:38744	5.21E-05	0.189	N/A		
2249069|F|014	G>A	3B	224721:15888	8.17E-05	0.177	gnl|UG|Ta#S61725485	Transmembrane protein, putative	TRIAE_CS42_3B_TGACv1_224721_AA0800650.1

### Gene identification

Significant SNP markers resulting from GWAS were mapped to the wheat unigene database. The identified unigenes were annotated based on the sequence comparisons using NCBI BLAST and EnsemblPlant databases. The first and second most significant SNP markers were associated with the unigenes TRIAE_CS42_5AL_TGACv1_376159_AA1232950 and gnl|UG|Ta#S52545076, respectively. The third and fourth SNPs respectively were associated with the genes TRIAE_CS42_2DS_TGACv1_179544_AA0607850 and TRIAE_CS42_3B_TGACv1_224721_AA0800650.1. The gene TRIAE_CS42_5AL_TGACv1_376159_AA1232950 is uncharacterized as judged from a lack of its functional annotation in wheat and other plant species. The unigene gnl|UG|Ta#S52545076 was 60% identical at the amino acid level with a gene in the *Tubulin* superfamily, *Tubulin* β*-1 chain*, of *Triticum urartu*. TRIAE_CS42_2DS_TGACv1_179544_AA0607850 showed 82% identity over 97% amino acid coverage with the Auxin-induced protein *5NG4* of *Aegilops tauschii*, whereas TRIAE_CS42_3B_TGACv1_224721_AA0800650.1 was annotated based on 51% amino acid identity and 97% coverage with a putative transmembrane protein of *Medicago truncatula* (Table 1).

## Discussion

From a larger set of 288 diverse bread wheat lines, we used 268 well-structured accessions to study the genetic association of cellulose content in wheat. The most appropriate model was selected to obtain a higher level of confidence in the association results. GWAS was conducted using Fixed and Random Model Circulating Probability Unification (FarmCPU); a new and more efficient recently developed method, which accounts for fixed and random effects to control false positives (Liu et al., 2016). Most of the GWAS mapping studies in wheat thus far have been conducted to identify genes or QTLs related to agronomic performance (Lopes et al., 2015; Jaiswal et al., 2016), grain yield (Sukumaran et al., 2015), or disease resistance (Kollers et al., 2013; Gurung et al., 2014). Cellulose is a major component of cell walls and a key determinant of mechanical strength of plant tissues (Appenzeller et al., 2004; Ching et al., 2006). The involvement of the *CesA* genes in cellulose synthesis is well-documented, and recently 22 *CesA* genes were reported in wheat (Kaur et al., 2016). These genes are differentially expressed in primary and secondary cell wall forming cells. Although, we identified 9 SNP markers in this study to be associated [−log10(*p*) = 7 to −log10(*p*) = 5] with cellulose content (Table S1), we were able to map only four of these to the wheat unigene database. A high marker density and population size in our study increased the confidence about these SNP associations (Wang et al., 2012). The genes associated with the cellulose content may contribute to its natural variation in wheat lines. The involvement of many genes other than *CesAs* in controlling cellulose synthesis supports our suggestion (Kotake et al., 2011).

Only a few studies have explored the genes other than *CesA* involved in the cellulose biosynthetic pathway (Porth et al., 2013; Slavov et al., 2014; Houston et al., 2015; Li et al., 2016). Recently in a GWA study in barley, a species syntenic to wheat, the association of several genes from the *Glycosyltransferases* and *Glycosylhydrolases* families was shown with the culm cellulose content (Houston et al., 2015). Similar to barley GWAS associations, our results also pointed to the involvement of the *GT* gene family in cellulose formation. We also identified some unique associations not reported in the barley study.

Our results pointed to the involvement of β*-tubulin* in the regulation of cellulose content. β*-tubulins* proteins form heterodimers with α*-tubulins* to form microtubules, which have long been known to guide the deposition of cellulose microfibrils in the cell wall in a helical pattern (Rao et al., 2016). Functional association of cortical microtubules with cellulose synthase complexes is well-documented (Paredez et al., 2006; Chan et al., 2007, 2010; Wightman and Turner, 2008; Crowell et al., 2009; Gutierrez et al., 2009).

Another important association in our study is for the Auxin-induced protein, *5NG4*. This gene is a member of the plant drug/metabolite exporter (P-DME; TC 2.A.7.4) family, also called WALLS ARE THIN1 (WAT1)-related proteins. Mutant studies in Arabidopsis helped demonstrate its involvement in secondary cell wall formation. Comparative transcriptomics and metabolomics demonstrated synchronized downregulation of the secondary cell wall *CesAs* (*CesA8, CesA7, and CesA4*) and auxin metabolism genes (auxin-responsive genes and auxin influx transporter genes) in *wat1* mutants (Ranocha et al., 2010). Higher expression of PIN-like auxin efflux carrier and auxin-induced protein *5NG4* genes in relation to both cell division and cell expansion was found in an expression profiling study of Chinese fir (*Cunninghamia lanceolata*; Qiu et al., 2013). With regard to the association of a putative transmembrane protein of unknown function with cellulose content in our study, several membrane proteins other than *CesA* are known to be involved in cellulose formation. We could not, however, assign a putative function to this protein. The additional genes we have reported in this study to be associated with cellulose content in the wheat culm are potential candidates for improving culm strength and the potential of wheat stover for biofuels by increasing the cellulose content.

## Conclusion

Cellulose content in wheat culms of 288 diverse cultivars varied widely. Genome-wide association analysis helped identify four genetic associations for cellulose content, which have the potential as molecular markers to manipulate cellulose content in wheat with the goal of improving culm strength and cellulosic biofuel production.

## Author contributions

SK performed all the experiments in the greenhouse and laboratory set up and conducted genetic, genomic, bioinformatics analyses, and wrote the manuscript. AM helped in writing and editing the manuscript. JS helped in troubleshooting, provided constructive comments, suggestions and financial support to conduct the experiments. KD provided the protocols for cellulose content analysis and thoroughly edited the manuscript. PV and SS provided the genotyping data, XZ, AM, and HD helped in the creating the SNP data and GWAS analysis. ZZ and KG provided their expert advice and suggestions to interpret the results.

### Conflict of interest statement

The authors declare that the research was conducted in the absence of any commercial or financial relationships that could be construed as a potential conflict of interest.
