# Supplementary material for: Genome-Wide Association Study Reveals Novel Genes Associated with Culm Cellulose Content in Bread Wheat (Triticum aestivum, L.)
Source: Front Plant Sci. 2017 Nov 6;8:1913. doi: 10.3389/fpls.2017.01913 (PMC5681534; doi:10.3389/fpls.2017.01913)
Supplement: Supplementary file 1 [file SupplementaryTables.docx]

**Table S1.** Sequences of SNPs significantly associated with stem cellulose content variation.

| **SNP ID** | **Allele** |
| --- | --- |
| 1096787\|F\|040 | CTTGCCACGACCGATTATCACCAACGACTGACAAGCCACGCCCCATTTTGGGCTGCCCTGCGCG |
| 1018641\|F\|062 | TCCAGCAACAAATGACTTGGTTGTATAGTCCGTAGGCACATCGGGAGTTGTTTCTTGTTGTAGT |
| 100315676\|F\|050 | CTCATGTCGTCTAGCACGTCGAACACCTCGGAGATGAGCCCCGTGTGGTCGGCGCTCGTCAGCT |
| 1080815\|F\|044 | CAGTTACACTAGAGAGTTGGATAAAAGCTTCTGCTATTTTCAAAGAAAATCGGTCACTTTGGAG |
| 3026141\|F\|05 | ACCGTGCGTGCCCGTGCACGTGTCCGTGCCGCCCGAGATCGGAAGAGCGGTTCAGCAGGAATGC |
| 1018617\|F\|035 | GATGCTCATGGTGATGGCTCCCCCCAGGCACAGAAGGGTCCCCACTATCTTGGCTCTTGTGTAC |
| 1245047\|F\|039 | GCAAGCTCTTGGGTTTCTTGGTTTCTAACAGAGGCATTGAAGCTAACCCGAGATCGGAAGAGCG |
| 1069330\|F\|06 | TTTTTCCAAAATTATGGTATTTTCTCTGCTTATAAAAAAGAACCCCCGACCTCTTTTTTAAAAC |
| 2249069\|F\|014 | CGTCCTCATGTGCGCGCTGCTCTACTTCCTCGACACCTCCGCGGACTACGCCAAGGGGATACAG |

**Table S2.** Percent variation for cellulose content among 288 diverse wheat lines along with their countries of origin.

| **Line No.** | **Name** | **Country of origin** | **% Cellulose (w/w)** |
| --- | --- | --- | --- |
| KSG001 | GABO 60 | Mexico | 46.70 |
| KSG002 | NACOZARI F 76 | [Mexico](http://www.ars-grin.gov/cgi-bin/npgs/html/acchtml.pl?1414930) | 43.06 |
| KSG003 | YECORA ROJO 76 | [Mexico](http://www.ars-grin.gov/cgi-bin/npgs/html/acchtml.pl?1414930) | 41.58 |
| KSG004 | ANNAPURNA 1 | Nepal, India | 47.04 |
| KSG005 | KLEIN DRAGON | Argentina | 45.14 |
| KSG006 | MEXIPAK65 | Pakistan | 43.48 |
| KSG007 | BLUEBIRD 15 | Mexico | 43.02 |
| KSG008 | ABU GHRAIB#3 | Iraq | 45.29 |
| KSG009 | FAISLABAD 83 | Pakistan | 48.26 |
| KSG010 | PUNJAB 88 | Pakistan | 47.78 |
| KSG011 | SAN CAYETANO S 97 | Mexico | 40.35 |
| KSG012 | BR 18 | Brazil | 43.04 |
| KSG013 | KENYA KWALE | Kenya | 44.92 |
| KSG014 | TEMPORALERA M87 | Kansas | 42.12 |
| KSG015 | ESTANZUELA PELON 90 | Uruguay | 43.81 |
| KSG016 | CHAM 6 | Syria | 44.95 |
| KSG017 | TINAMOUII | Mexico | 42.13 |
| KSG018 | ARIVECHI M 92 | Mexico | 46.23 |
| KSG019 | YAQUI 50 | Mexico | 45.87 |
| KSG020 | NARINO 59 | [Colombia](http://www.ars-grin.gov/cgi-bin/npgs/html/acchtml.pl?1065708) | 47.53 |
| KSG021 | PENJAMO T 62 | Mexico | 44.09 |
| KSG022 | PITIC62 | Mexico | 43.40 |
| KSG023 | CRESPO | Colombia | 43.45 |
| KSG024 | NADADORES M 63 | [Mexico](http://www.ars-grin.gov/cgi-bin/npgs/html/acchtml.pl?1414930) | 43.27 |
| KSG025 | SONORA 64 | Mexico | 43.45 |
| KSG026 | INIA F66 | Mexico | 42.62 |
| KSG027 | BAJIO | Mexico | 47.57 |
| KSG028 | KALYANSONA | India | 44.83 |
| KSG029 | SAFED LERMA | India | 43.51 |
| KSG030 | SONALIKA | India | 43.67 |
| KSG031 | CALIDAD | Argentina | 43.06 |
| KSG032 | UP301 | India | 35.07 |
| KSG033 | POTAM S 70 | Mexico | 41.06 |
| KSG034 | MARCOS JUAREZ INTA | [Argentina](http://www.ars-grin.gov/cgi-bin/npgs/html/acchtml.pl?1454651) | 46.21 |
| KSG035 | TANORI F 71 | Mexico | 45.45 |
| KSG036 | ARZ | Lebanon | 44.91 |
| KSG037 | JUPATECO F 73 | Mexico | 43.09 |
| KSG038 | MAYA 74 | [Guatemala](http://www.ars-grin.gov/cgi-bin/npgs/html/acchtml.pl?1414753) | 44.13 |
| KSG039 | SALAMANCA 75 | Spain | 44.63 |
| KSG040 | LIESBECK | South Africa | 43.34 |
| KSG041 | PAVON F 76 | Mexico | 42.55 |
| KSG042 | SAKHA 8 | Egypt | 44.00 |
| KSG043 | CHIVITO | Australia | 39.88 |
| KSG044 | HERMOSILLO M77 | Mexico | 36.17 |
| KSG045 | SERI M 82 | Mexico | 44.15 |
| KSG046 | UP262 | Nepal, India | 43.92 |
| KSG047 | BAHAWALPUR 79 | Pakistan | 43.74 |
| KSG048 | SAKHA 69 | Egypt | 44.43 |
| KSG049 | HARTOG | Australia | 43.74 |
| KSG050 | PIRSABAK 85 | Pakistan | 44.80 |
| KSG051 | GONEN | Turkey | 46.36 |
| KSG052 | RAYON F 89 | Mexico | 41.49 |
| KSG053 | NESSER | Jordan | 40.51 |
| KSG054 | ICA YACUANQUER | Colombia | 43.72 |
| KSG055 | TIA.1 | Mexico | 43.22 |
| KSG056 | BORLAUG M 95 | Mexico | 43.14 |
| KSG057 | PBW343 | India | 42.65 |
| KSG058 | INIFAP M 97 | Chile | 42.22 |
| KSG059 | TOBARITO M 97 | Mexico | 42.83 |
| KSG060 | GRANERO INTA | Argentina | 42.20 |
| KSG061 | PROINTA OASIS | Argentina | 45.83 |
| KSG062 | ITAPUA 40-OBLIGADO | Paraguay | 44.87 |
| KSG063 | KLEIN DRAGON | Argentina | 43.50 |
| KSG064 | BAW898 | Bangladesh | 41.62 |
| KSG065 | CUMHURIYET 75 | Turkey | 39.23 |
| KSG066 | MILLALEAU INIA | Chile | 42.16 |
| KSG067 | IAN 8-PIRAPO | Turkey | 42.47 |
| KSG068 | PAVON | Mexico | 44.80 |
| KSG069 | POINTA FEDERAL | Argentina | 44.12 |
| KSG070 | SONALIKA | Punjab, India | 42.04 |
| KSG071 | ANDES-56 | Colombia | 44.15 |
| KSG072 | SARIAB-92 | Pakistan | 42.16 |
| KSG073 | OROFEN 60 | Chile | 40.17 |
| KSG074 | LERMA ROJO 64 | Mexico | 41.70 |
| KSG075 | V-17 | Mexico | 45.89 |
| KSG076 | PJ62/GB55 | Mexico | 46.46 |
| KSG077 | ZAMINDAR 80 | Pakistan | 36.97 |
| KSG078 | PAKISTAN 81 | Pakistan | 42.63 |
| KSG079 | CORDILLERA 3 | Paraguay | 45.06 |
| KSG080 | IDAHO 61M3404 | Idaho | 46.97 |
| KSG081 | IDAHO 62M9-224 | Idaho | 43.44 |
| KSG082 | LEMHI 66 | Idaho | 47.83 |
| KSG083 | 64AB9405 | ID | 43.04 |
| KSG084 | TWIN | Idaho | 41.84 |
| KSG085 | OWENS | Idaho | 44.57 |
| KSG086 | IDO190 | Idaho | 44.84 |
| KSG087 | IDO232 | Idaho | 42.35 |
| KSG088 | COPPER | Idaho | 42.16 |
| KSG089 | VANDAL | Idaho | 45.68 |
| KSG090 | IDAHO 266 | Idaho | 41.43 |
| KSG091 | WHITEBIRD | Idaho | 44.70 |
| KSG092 | FREX | Indiana | 41.80 |
| KSG093 | II-53-521 | Minnesota | 47.65 |
| KSG096 | II-55-1 | Minnesota | 39.91 |
| KSG097 | II-58-60 | Minnesota | 45.51 |
| KSG098 | II-62-78 | Minnesota | 40.85 |
| KSG099 | MN 6616M | [Minnesota](http://www.ars-grin.gov/cgi-bin/npgs/html/acchtml.pl?1078669) | 41.97 |
| KSG100 | WHEATON | Minnesota | 43.64 |
| KSG101 | II-64-20 | Minnesota | 40.51 |
| KSG102 | MN 6898 | [Minnesota](http://www.ars-grin.gov/cgi-bin/npgs/html/acchtml.pl?1414636) | 42.28 |
| KSG103 | VANCE | Minnesota | 43.07 |
| KSG104 | NORM | Minnesota | 47.17 |
| KSG105 | VERDE | Minnesota | 38.19 |
| KSG106 | MCVEY | [Nebraska](http://www.ars-grin.gov/cgi-bin/npgs/html/acchtml.pl?1397843) | 47.63 |
| KSG107 | JUSTIN | North Dakota | 49.32 |
| KSG108 | ND 202-2 | North Dakota | 48.39 |
| KSG109 | ND 271 | [North Dakota](http://www.ars-grin.gov/cgi-bin/npgs/html/acchtml.pl?1064009) | 49.82 |
| KSG110 | ND 229-1 | North Dakota | 49.86 |
| KSG111 | ND 287 | [North Dakota](http://www.ars-grin.gov/cgi-bin/npgs/html/acchtml.pl?1064009) | 46.92 |
| KSG112 | FORTUNA | North Dakota | 49.51 |
| KSG113 | LEEDS | North Dakota | 45.79 |
| KSG114 | ND 59-120A | North Dakota | 44.31 |
| KSG115 | ND 407 | North Dakota | 45.67 |
| KSG116 | WALDRON | North Dakota | 40.84 |
| KSG117 | ND 22 | North Dakota | 47.79 |
| KSG118 | ND 66 | North Dakota | 42.41 |
| KSG119 | CI014952 | North Dakota | 47.55 |
| KSG120 | CI014953 | North Dakota | 44.39 |
| KSG121 | ROLETTE | North Dakota | 45.66 |
| KSG122 | D 6647 | North Dakota | 45.28 |
| KSG123 | ND 467 | North Dakota | 48.81 |
| KSG124 | ND 476 | North Dakota | 39.68 |
| KSG125 | ELLAR | North Dakota | 47.47 |
| KSG126 | EDMORE | North Dakota | 47.67 |
| KSG127 | COTEAU | North Dakota | 46.89 |
| KSG128 | D804 | North Dakota | 41.71 |
| KSG129 | MONROE | [North Dakota](http://www.ars-grin.gov/cgi-bin/npgs/html/acchtml.pl?1373234) | 44.96 |
| KSG130 | D7925 | North Dakota | 46.32 |
| KSG131 | ND 13-137 | North Dakota | 46.89 |
| KSG132 | AMIDON | North Dakota | 47.91 |
| KSG133 | MUNICH | North Dakota | 43.58 |
| KSG134 | PIERCE | North Dakota | 44.66 |
| KSG135 | ND 2710 | [North Dakota](http://www.ars-grin.gov/cgi-bin/npgs/html/acchtml.pl?1064009) | 43.36 |
| KSG136 | STW 598874 | Oklahoma | 46.21 |
| KSG137 | YSCA-1 | Oklahoma | 42.49 |
| KSG139 | SEL. 90 | Washington | 46.36 |
| KSG140 | WA 6101 | Washington | 40.09 |
| KSG141 | WA 7175 | Washington | 46.07 |
| KSG142 | SPILLMAN | Washington | 43.59 |
| KSG143 | ARS95 451 | Washington | 45.09 |
| KSG144 | ARS95 457 | Washington | 48.02 |
| KSG145 | EDEN | Washington | 43.61 |
| KSG146 | ALPOWA | Washington | 43.44 |
| KSG147 | ALTURAS | Idaho | 43.76 |
| KSG148 | CHALLIS | Montana | 44.41 |
| KSG149 | EDWALL | Washington | 43.37 |
| KSG151 | JUBILEE | Idaho | 47.38 |
| KSG152 | VANNA | ARIZONA | 47.11 |
| KSG153 | TARA 2002 AKA TARA | Washington | 41.09 |
| KSG154 | SCARLET | Washington | 46.84 |
| KSG155 | JEFFERSON | Idaho | 43.35 |
| KSG156 | HOLLIS | Washington | 46.53 |
| KSG157 | CALORWA | Washington | 45.43 |
| KSG158 | ZAK | Washington | 50.19 |
| KSG159 | WAWAWAI | Washington | 46.65 |
| KSG160 | CENTENNIAL | Idaho | 45.11 |
| KSG161 | MACON | Washington | 49.06 |
| KSG162 | LOLO | Idaho | 45.98 |
| KSG163 | KLASIC | Nebraska | 48.78 |
| KSG164 | IDO377S | Washington | 45.83 |
| KSG165 | YECORA ROJO | Mexico | 47.68 |
| KSG166 | SAXON | Colorado | 43.87 |
| KSG167 | NEWANA | Montana | 45.93 |
| KSG168 | URQUIE | Washington | 45.29 |
| KSG169 | RUSHMORE | South Dakota | 52.13 |
| KSG170 | RAMONA | California | 47.26 |
| KSG171 | HARD FEDERATION AKA PI041079 | Australia | 46.43 |
| KSG172 | REDCHAFF | Washington | 48.07 |
| KSG173 | SELKIRK | Canada | 47.15 |
| KSG176 | SAUNDERS | Canada | 47.22 |
| KSG177 | LEE | Minnesota | 50.12 |
| KSG178 | PEAK | Idaho | 46.80 |
| KSG179 | AKA PROBRAND 751 | Nebraska | 46.81 |
| KSG180 | WADUAL | Washington | 46.88 |
| KSG181 | WAKANZ | Washington | 50.51 |
| KSG182 | CANTHATCH | Canada | 47.41 |
| KSG183 | CONLEY | North Dakota | 50.25 |
| KSG184 | PEAK 72 | Idaho | 50.30 |
| KSG185 | PROSPUR | Minnesota | 49.31 |
| KSG186 | KITT AKA PI518818 | Minnesota | 40.43 |
| KSG187 | WAMPUM | Washington | 46.33 |
| KSG188 | WALLADAY | Washington | 43.50 |
| KSG189 | PONDERA | Montana | 43.95 |
| KSG190 | STERLING | Idaho | 42.86 |
| KSG191 | MCKAY | [Idaho](http://www.ars-grin.gov/cgi-bin/npgs/html/acchtml.pl?1080438) | 47.19 |
| KSG192 | WAID | Washington | 45.85 |
| KSG194 | NORANA | Montana | 45.85 |
| KSG195 | OLAF | North Dakota | 43.50 |
| KSG196 | BORAH | Idaho | 47.91 |
| KSG197 | WAVERLY | Washington | 42.25 |
| KSG198 | TREASURE | Idaho | 43.74 |
| KSG199 | WESTBRED 906R | Arizona | 43.59 |
| KSG200 | WESTBRED 911 | Arizona | 43.17 |
| KSG201 | BLISS | Idaho | 48.89 |
| KSG202 | WARD | North Dakota | 44.00 |
| KSG203 | BOUNTY 208 | Colorado | 46.40 |
| KSG204 | ANZA | California | 47.21 |
| KSG205 | MORAN | [Idaho](http://www.ars-grin.gov/cgi-bin/npgs/html/acchtml.pl?1064653) | 48.77 |
| KSG206 | UNION | Oregon | 45.29 |
| KSG207 | UTAC | Utah | 44.46 |
| KSG208 | WHITE FIFE AKA PI061345 | Japan | 46.52 |
| KSG209 | WHITE MARQUIS | Minnesota | 43.86 |
| KSG210 | SEA ISLAND | Colorado | 48.51 |
| KSG211 | RUBY | Canada | 47.76 |
| KSG212 | RIVAL | North Dakota | 50.55 |
| KSG213 | LEMHI | Idaho | 46.40 |
| KSG214 | LITTLE CLUB | Oregon | 48.76 |
| KSG215 | MARFED | [Washington](http://www.ars-grin.gov/cgi-bin/npgs/html/acchtml.pl?1058207) | 49.91 |
| KSG216 | TOUSE | Utah | 47.86 |
| KSG217 | THATCHER | Minnesota | 48.18 |
| KSG218 | SUPREME | Canada | 48.16 |
| KSG219 | SPINKCOTA | South Dakota | 45.55 |
| KSG220 | SONORA | Mexico | 42.30 |
| KSG221 | GALGALOS AKA PI009872 | Armenia | 50.42 |
| KSG222 | FEDERATION 67 | Idaho | 47.80 |
| KSG223 | FEDERATION AKA PI041080 | Australia | 43.81 |
| KSG224 | REWARD | Canada | 48.19 |
| KSG225 | RESCUE | Canada | 48.80 |
| KSG226 | RELIANCE | Oregon | 52.02 |
| KSG227 | REGENT | Canada | 48.88 |
| KSG228 | RED BOBS | Canada | 50.14 |
| KSG229 | RAMONA 50 | California | 42.15 |
| KSG230 | ORFED | Washington | 43.57 |
| KSG231 | OREGON ZIMMERMAN | Oregon | 48.74 |
| KSG232 | ONAS 53 | California | 48.08 |
| KSG234 | MIDA | [North Dakota](http://www.ars-grin.gov/cgi-bin/npgs/html/acchtml.pl?1058636) | 50.03 |
| KSG235 | MARQUIS | [Canada](http://www.ars-grin.gov/cgi-bin/npgs/html/acchtml.pl?1025379) | 47.74 |
| KSG236 | PACIFIC BLUESTEM | Oregon | 48.55 |
| KSG237 | PACIFIC BLUESTEM 37 | California | 48.18 |
| KSG238 | PILOT | North Dakota | 48.54 |
| KSG239 | PREMIER | North Dakota | 48.32 |
| KSG240 | ALLEN | Washington | 45.65 |
| KSG241 | AWNED ONAS | California | 46.61 |
| KSG242 | BAART EARLY SELECTION | California | 43.80 |
| KSG243 | CANADIAN RED | California | 44.43 |
| KSG244 | CADET | North Dakota | 43.74 |
| KSG245 | BLUECHAFF | Oregon | 41.85 |
| KSG246 | BIG CLUB | Oregon, California | 44.45 |
| KSG247 | HARD FEDERATION (-31) | Oregon | 40.96 |
| KSG248 | HENRY | Wisconsin | 45.21 |
| KSG249 | HOPE | South Dakota | 48.84 |
| KSG250 | HYBRID 63 | Washington | 35.98 |
| KSG252 | KINNEY | Oregon | 41.70 |
| KSG253 | KENHI | Canada | 47.61 |
| KSG254 | CERES | North Dakota | 48.80 |
| KSG255 | WESTBRED EXPRESS | Arizona | 37.88 |
| KSG256 | LAGODA | Russian | 45.89 |
| KSG257 | FLOMAR | Washington | 49.09 |
| KSG258 | HYBRID 123 | Washington | 36.31 |
| KSG259 | DICKLOW | Utah | 47.27 |
| KSG260 | GYPSUM | Colorado | 46.78 |
| KSG261 | HYPER | Washington | 46.61 |
| KSG262 | IDAED | Idaho | 45.04 |
| KSG263 | INDIAN | Idaho | 45.02 |
| KSG264 | BAART 46 | California | 51.13 |
| KSG265 | NEW ZEALAND | Nevada | 41.83 |
| KSG267 | PILCRAW | California | 44.72 |
| KSG268 | RINK | Oregon | 47.30 |
| KSG269 | SURPRISE | Vermont | 46.20 |
| KSG270 | WHITE FEDERATION | Australia | 40.98 |
| KSG271 | BUNYIP | Australia | 43.41 |
| KSG272 | CURRAWA | Australia | 44.45 |
| KSG273 | WILBUR | Oregon | 43.52 |
| KSG274 | EARLY BAART | California | 45.35 |
| KSG275 | MAJOR | Australia | 46.16 |
| KSG276 | LEMHI 53 | Idaho | 43.78 |
| KSG277 | SPRINGFIELD | Idaho | 46.77 |
| KSG278 | FIELDER | Idaho | 45.70 |
| KSG279 | FIELDWIN | Idaho | 43.40 |
| KSG282 | SCHLANSTEDT | New York | 44.55 |
| KSG283 | PRESTON | Canada | 46.26 |
| KSG284 | CHINOOK | North Dakota | 42.48 |
| KSG285 | MANITOU | [Canada](http://www.ars-grin.gov/cgi-bin/npgs/html/acchtml.pl?1064766) | 44.93 |
| KSG286 | RED RIVER 68 | California | 43.17 |
| KSG287 | ERA | Minnesota | 40.37 |
| KSG288 | BOUNTY 309 | Colorado | 42.15 |
| KSG289 | WINSOME | Oregon | 42.23 |
| KSG290 | AIM | ARIZONA | 44.88 |
| KSG291 | BRONZE CHIEF | USA | 41.58 |
| KSG292 | KODIAK DWARF | USA | 48.34 |
| KSG293 | KUBANKA | USA | 43.58 |
| KSG294 | KAHLA | Algeria | 48.80 |
| KSG295 | SENTRY | North Dakota | 47.97 |
| KSG297 | WELLS | North Dakota | 50.19 |
| KSG298 | WANDELL | Washington | 49.60 |
| KSG299 | PRODURA | Minnesota | 45.86 |
| KSG301 | WL 444 | - | 45.29 |
| KSG302 | POMERELLE | Idaho | 47.03 |
